# Antibiotic Treatments for *Clostridium difficile* Infection Are Associated with Distinct Bacterial and Fungal Community Structures

**DOI:** 10.1128/mSphere.00572-17

**Published:** 2018-01-10

**Authors:** Regina Lamendella, Justin R. Wright, Jada Hackman, Christopher McLimans, David R. Toole, William Bernard Rubio, Rebecca Drucker, Hoi Tong Wong, Kate Sabey, John P. Hegarty, David B. Stewart

**Affiliations:** a Juniata College, Biology Department, Huntingdon, Pennsylvania, USA; b Department of Surgery, Division of Colon and Rectal Surgery, The Pennsylvania State University, College of Medicine, Hershey, Pennsylvania, USA; Arizona State University

**Keywords:** *Clostridium difficile* infection, antibiotic treatments, intestinal dysbiosis

## Abstract

*Clostridium difficile* infection (CDI) is the most common nosocomial infection in the United States, being associated with high recurrence and persistence rates. Though the relationship between intestinal dysbiosis and CDI is well known, it is unclear whether different forms of dysbiosis may potentially affect the course of CDI. How this is further influenced by *C. difficile*-directed antibiotics is virtually uninvestigated. In this study, diarrheal stool samples were collected from 20 hospitalized patients, half of whom were confirmed to have CDI. Analyzing tissue *ex vivo* and in duplicate, CDI and non-CDI fecal samples (*n* = 176) were either not antibiotic treated or treated with metronidazole, vancomycin, or fidaxomicin, the three most common CDI therapies. The microbial community composition, interactions, and predicted metabolic functions were assessed by 16S rRNA gene and internal transcribed spacer sequencing, bipartite network analysis, and phylogenetic investigation of communities by reconstruction of unobserved states. Our results demonstrate that while all *C. difficile*-directed antibiotics were associated with similar reductions in alpha diversity, beta diversity significantly differed on the basis of the particular antibiotic, with differentiating relative abundances of bacterial and fungal assemblages. With the exception of fidaxomicin, each antibiotic was associated with the emergence of potentially pathogenic fungal operational taxonomic units, with predicted bacterial functions enriched for xenobiotic metabolism that could perpetuate the dysbiosis driving CDI. Toxin-independent mechanisms of colitis related to the relative abundance of pathogenic bacteria and fungi were also noted. This study suggests that a transkingdom interaction between fungi and bacteria may be important in CDI pathophysiology, including being a factor in the historically high persistence and recurrence rates associated with this disease.

**IMPORTANCE** Using human fecal samples and including sequencing for both bacterial and fungal taxa, this study compared the conventional antibiotics used to treat *C. difficile* infection (CDI) from the perspective of the microbiome, which is particularly relevant, given the relationship between dysbiotic states and the development of CDI. Sequencing and imputed functional analyses suggest that *C. difficile*-directed antibiotics are associated with distinct forms of dysbiosis that may be influential in the course of CDI. Further, a role for fungal organisms in the perpetuation of the causal dysbiosis of CDI is discussed, suggesting a previously unappreciated, clinically relevant transkingdom interaction that warrants further study.

## Abstract (precis)

Using human fecal samples and including sequencing for both bacterial and fungal taxa, this study compared the conventional antibiotics used to treat *C. difficile* infection (CDI) from the perspective of the microbiome, which is particularly relevant, given the relationship between dysbiotic states and the development of CDI. Sequencing and imputed functional analyses suggest that *C. difficile*-directed antibiotics are associated with distinct forms of dysbiosis that may be influential in the course of CDI. Further, a role for fungal organisms in the perpetuation of the causal dysbiosis of CDI is discussed, suggesting a previously unappreciated, clinically relevant transkingdom interaction that warrants further study.

## INTRODUCTION

In its most common form, *Clostridium difficile* infection (CDI) is causally associated with an antibiotic-induced disturbance (1, 2) of the gut microbial community structure associated with a normal state of health. Studies focused on metagenomics have characterized the dysbiosis of CDI as being associated with a significant reduction in taxa belonging to the *Bacteroidetes* and *Firmicutes* phyla, with a concomitant relative abundance of *Proteobacteria* (3, 4). This compositional change in the gut bacterial community results in other compositional changes, such as a prominent reduction in secondary bile acids (5, 6) leading to germination of *C. difficile* spores, thus promoting higher rates of recurrent infection (7). Under ambient conditions that are hostile to many nonpathogenic bacteria, *C. difficile* demonstrates a strong selective advantage toward mucosal adherence and colonization (8, 9), thus occupying a niche that allows it to thrive (10).

The goal of *C. difficile*-directed antibiotics is to eradicate CDI while limiting disturbances of other bacterial taxa. Three antibiotics are commonly used for the treatment of CDI: metronidazole (11), vancomycin (12), and fidaxomicin (13). Of these, fidaxomicin and its major active metabolite (OP-1118) have the narrowest reported spectrum of antibacterial activity (14). While these drugs have a narrower spectrum of activity, all of these antibiotics have mechanisms of action that necessarily lead to off-target effects on non-*C. difficile* bacteria that become collateral damage of conventional antibiotic therapy. Paradoxically, conventional antibiotics for CDI may therefore sustain a dysbiosis (15) potentially sufficient to perpetuate the very infection these drugs are administered to treat.

Two clinically relevant yet unanswered questions are whether different *C. difficile*-directed antibiotics create distinct forms of antibiotic-induced dysbiosis and whether the microbial community structures that characterize these posttreatment gut ecologies are equally detrimental to a patient’s health. Two related and virtually unstudied questions are how the gut mycobiome might contribute to the pathogenesis (16) of this bacterial infection and whether different *C. difficile*-directed antibiotics lead to the relative abundance of distinct fungal organisms that might potentially influence the course of CDI. Distinct from issues of efficacy, which is the usual focus of antibiotic therapy, there has been very little comparison of *C. difficile*-directed therapies, especially those using human tissue, to assess whether one of these drugs might result in a microbial composition closer to a eubiotic state, which might be a less deleterious form of gut ecological disturbance. In such an instance, apart from issues of cost, clinicians might select one drug over others on the basis of its impact on the microbiome, an important consideration given that changes in bacterial population diversity and density are key elements of the etiology of CDI.

The present study obtained patient-derived *C. difficile*-positive and -negative diarrheal stool samples treated *ex vivo* with each of three *C. difficile*-directed antibiotics to identify whether any of these drugs produce distinct microbial community structures that might suggest an influence on treatment success. Sequencing of fungal taxa was included to further investigate a role for the mycobiome in CDI.

## RESULTS

The CDI patient cohort, with a mean age of 70.4 ± 18.4 years, was younger than the non-CDI cohort, with a mean age of 56 ± 19.0 years (*t* test, *P* = 0.05). There was no statistically significant difference in the number of chronic comorbidities shared by the two patient cohorts, with the most common chronic conditions being hypertension and non-insulin-dependent diabetes mellitus.

### Bacterial community structure comparisons.

As demonstrated in prior studies, untreated CDI was associated with a significant reduction in alpha diversity across all samples (nonparametric *t* test with Monte Carlo permutations, CDI, 90.67 ± 49.25 observed species versus non-CDI, 426 ± 724 observed species; two-sample *t* test with Monte Carlo permutations test statistic = 6.51 [*P* < 0.001]). When the bacterial species richness of the antibiotic treatment groups was evaluated, it was found to be elevated across all respective non-CDI cohorts when considering observed species and phylogenetic diversity (PD) whole-tree metrics in comparison to respective CDI cohorts (see Fig. S1 in the supplemental material). Only CDI cohorts with fidaxomicin and metronidazole treatment had significantly less species richness than respective non-CDI cohorts (fidaxomicin, PD whole-tree test statistic = 6.618 [*P* < 0.028], observed species test statistic = −5.30 [*P* < 0.028]; metronidazole, PD whole-tree test statistic = 6.969 [*P* < 0.028], observed species test statistic = −6.00 [*P* < 0.028]).

10.1128/mSphere.00572-17.1 FIG S1  Alpha diversity boxplots were generated in QIIME 1.9.0 to compare PD whole-tree (left) and observed species (right) metrics between sample antibiotic cohorts further stratified by disease status (CDI versus non-CDI). Boxplots are colored by respective antibiotic treatments. A horizontal red line indicates the median metric value for each respective cohort. Quantitative outputs of two-sample *t* tests with Monte Carlo permutations of CDI and non-CDI samples from each treatment cohort are displayed below the respective boxplots. Download FIG S1, JPG file, 1.7 MB. Copyright © 2018 Lamendella et al. 2018 Lamendella et al. This content is distributed under the terms of the Creative Commons Attribution 4.0 International license.

**FIG 1:**
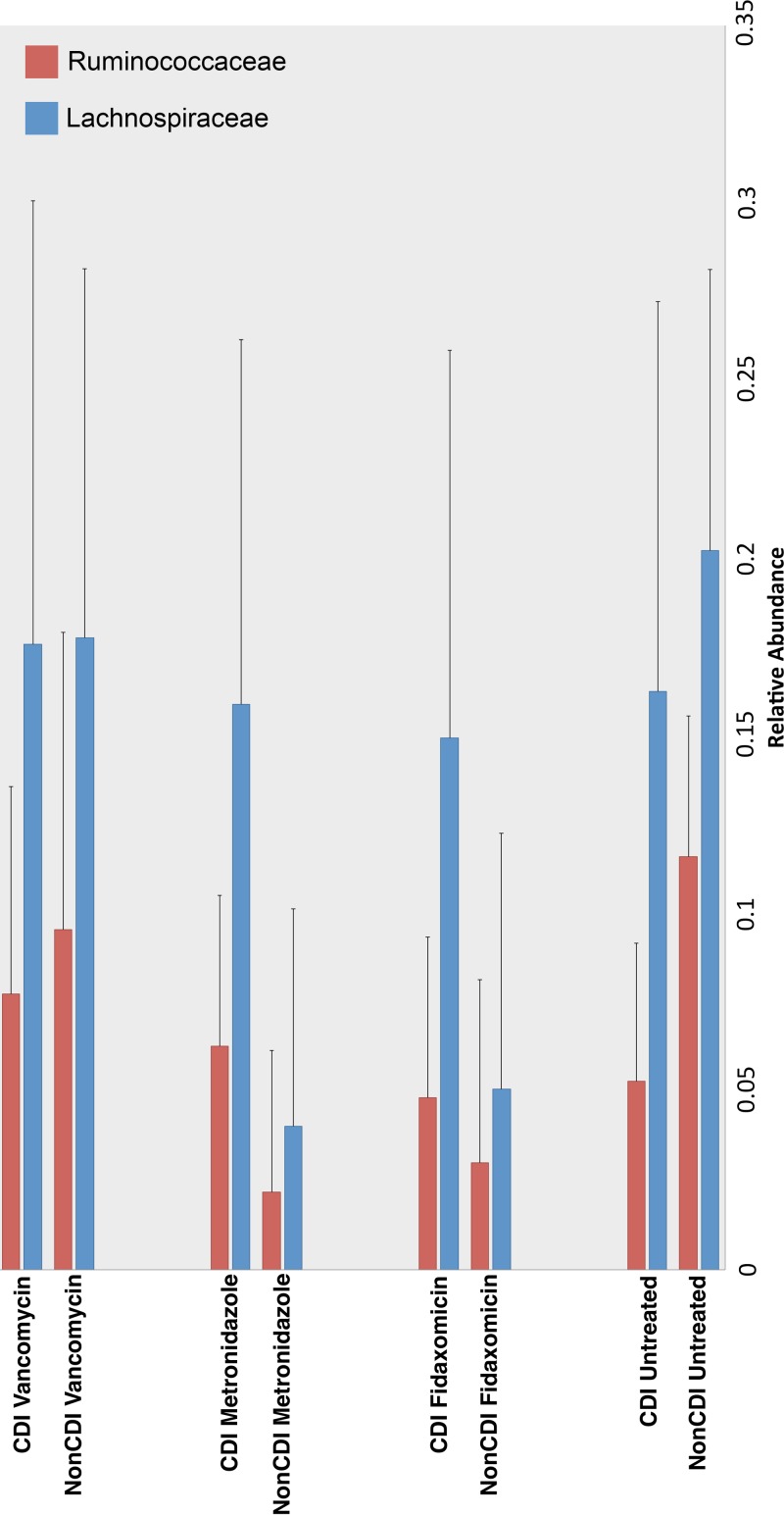
Family level bar plots generated in QIIME 1.9.0 display differences in CSS-normalized relative abundances of commensal butyrogenic bacteria in each respective treatment cohort (CDI versus non-CDI). Greater abundances of both *Ruminococcaceae* and *Lachnospiraceae* in the untreated non-CDI cohort than in the untreated CDI samples. An increase in *Lachnospiraceae* and *Ruminococcaceae* abundance can be observed in the CDI cohort when considering the fidaxomicin and metronidazole treatment cohorts. In the vancomycin treatment cohort, a 1.9% decrease in *Ruminococcaceae* can be observed in the CDI cohort, whereas *Lachnospiraceae* did not differ substantially (±0.001%) between disease statuses.

When considering untreated samples, a significant decrease in butyrogenic organisms such as *Lachnospiraceae* and *Ruminococcaceae* was observed in CDI patients (11.6 and 5.3%, respectively) compared to the control cohort (20.2 and 16.2%, respectively) of patients with diarrhea who tested negative for CDI (Kruskal-Wallis test, linear discriminant analysis [LDA] cutoff score of >2.0 [*P* < 0.05]) (Fig. 1). Interestingly, when CDI and non-CDI cohorts of patients receiving fidaxomicin and metronidazole were compared, a higher relative abundance of the families *Lachnospiraceae* and *Ruminococcaceae* was observed in the CDI cohort. In the vancomycin-treated samples, there was a lower abundance of *Ruminococcaceae* (−1.9%) in the CDI cohort, whereas the abundance of *Lachnospiraceae* did not differ substantially (±0.001%).

**FIG 2:**
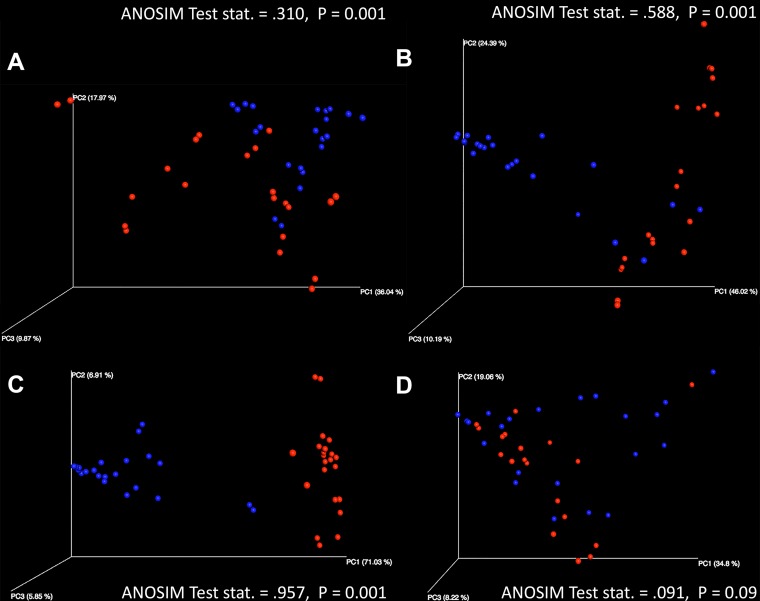
Three-dimensional principal-coordinate (PC) analysis plots were generated in QIIME 1.9.0 and visualized with EMPeror to display overall weighted UniFrac distances between CDI and non-CDI samples in each treatment cohort (A, untreated; B, fidaxomicin; C, metronidazole; D, vancomycin). Here, we can visualize significant differential clustering between disease statuses when considering untreated, fidaxomicin-treated, and metronidazole-treated samples (ANOSIM *P* = 0.001). Vancomycin-treated samples, however, do not yield significantly differential clustering (ANOSIM *P* = 0.09).

Principal-coordinate analysis of weighted UniFrac distances demonstrated significant clustering of samples among differential antibiotic-treated sample cohorts. When considering fidaxomicin-treated, metronidazole-treated, and untreated cohorts, significantly differential clustering was observed in CDI and non-CDI samples (Fig. 2A to C; analysis of similarity [ANOSIM] test statistics = 0.588 [*P* = 0.001], 0.957 [*P* = 0.001], and 0.310 [*P* = 0.001], respectively). CDI and non-CDI samples that underwent vancomycin treatment did not exhibit significantly different overall community structures (Fig. 2D; ANOSIM test statistic = 0.091 [*P* = 0.09]).

**FIG 3:**
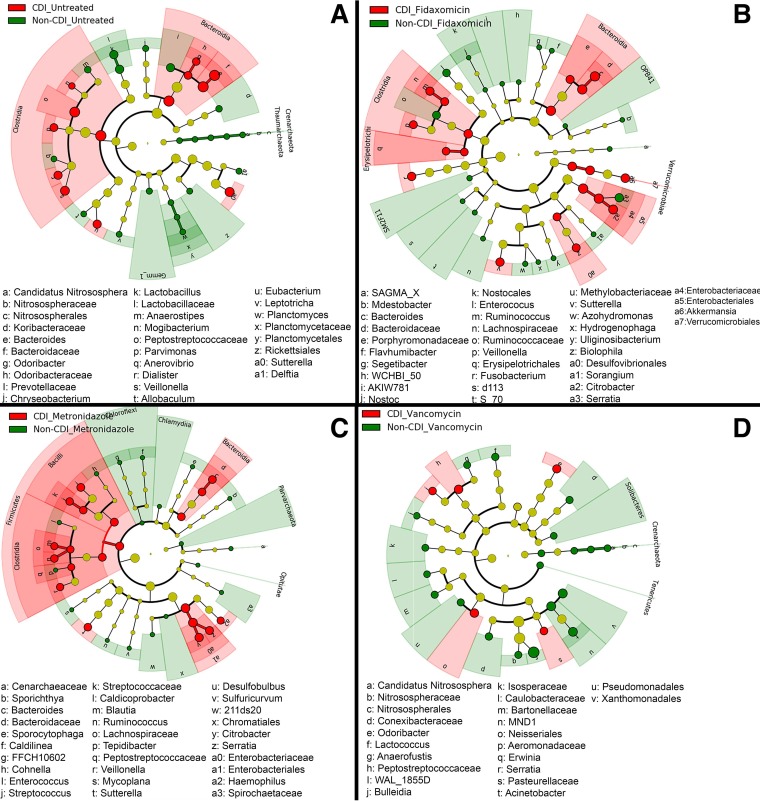
Cladogram plots were generated in Galaxy to visualize significantly enriched bacterial taxa identified in CDI and non-CDI samples considering each treatment cohort separately (A, untreated; B, fidaxomicin; C, metronidazole; D, vancomycin).

Paired LDA effect size (LEfSe) analyses were performed to identify significant increases in the relative abundance (LDA cutoff score of >2.0 [*P* < 0.05]) of bacterial taxa in samples paired by untreated and antibiotic-treated status (Fig. 3). For untreated stool samples, the most significantly enriched bacterial sequences in the non-CDI group were *Leptotrichia*, taxa of the phylum *Planctomyces*, and archaea of the family *Nitrososphaeraceae*; in the untreated CDI cohort, members of the classes *Clostridia* and *Bacteroidia* were strongly enriched. In metronidazole-treated samples, the non-CDI cohort demonstrated comparable abundances of *Chlamydia*, *Desulfobulbus*, and *Sporichthya* and archaeal *Parvarchaea* taxa. In treated CDI samples, metronidazole was associated with relative abundances of *Enterobacteriales* and *Citrobacter* organisms. Following vancomycin treatment, non-CDI samples demonstrated a higher abundance of sequences matching *Tenericutes* and the *Serratia* and *Erwinia* genera, the latter being members of the family *Enterobacteriaceae*. In CDI samples, vancomycin treatment was associated with relative abundances of *Peptostreptococcaceae*, *Neisseriales*, and the genus *Odoribacter*. Following fidaxomicin treatment, non-CDI stool samples demonstrated relative abundances of members of the genera *Serratia* and *Hydrogenophaga*, as well as the family *Ruminococcaceae*; in the CDI cohort, members of the order *Enterobacteriales* and the class *Bacteroidia* were more abundant (Fig. S2).

10.1128/mSphere.00572-17.2 FIG S2  LEfSe plots were generated to display biomarker bacterial taxa driving shifts in microbial community structure between CDI and non-CDI samples in untreated (A), fidaxomicin-treated (B), metronidazole-treated (C), and vancomycin-treated (D) samples. Download FIG S2, TIF file, 0.9 MB. Copyright © 2018 Lamendella et al. 2018 Lamendella et al. This content is distributed under the terms of the Creative Commons Attribution 4.0 International license.

Predictive functional profiling with phylogenetic investigation of communities by reconstruction of unobserved states (PICRUSt) was utilized to evaluate the gene families enriched in a given community by bacteria or archaea identified in the 16S rRNA gene data set. Among samples not treated with antibiotics, pathways for lipopolysaccharide (LPS) synthesis and metabolism were only enriched in CDI samples (Fig. S3). The CDI cohort not treated with antibiotics demonstrated predicted enrichment of pathways associated with arachidonic acid metabolism, folate biosynthesis, novobiocin synthesis, and sulfur relay systems. Following metronidazole treatment, non-CDI samples demonstrated predicted enrichment of pathways associated with xenobiotic degradation and metabolism and lipid, fatty acid, tryptophan, and butyrate metabolism; among CDI samples, metronidazole treatment was associated with predicted pathways for LPS synthesis, as well as metabolism of glycans and secondary metabolites. With vancomycin treatment, non-CDI samples demonstrated a greater abundance of starch and sucrose metabolism pathways, while CDI samples demonstrated predicted enrichment of LPS synthesis and metabolism pathways. In both CDI and non-CDI samples, the use of vancomycin was associated with enrichment of isoquinoline production. Among fidaxomicin-treated samples, non-CDI samples demonstrated predicted enrichment of pathways for xenobiotic degradation and metabolism, as well as tryptophan and fatty acid metabolism; in CDI samples, secondary metabolite metabolism, glycan biosynthesis and metabolism, and LPS pathways were enriched. The weighted nearest sequenced taxon index (NSTI) scores summarizing the extent to which taxa were related to sequenced genomes were ≤0.12.

10.1128/mSphere.00572-17.3 FIG S3  LEfSe plots were generated to display significantly enriched (LDA cutoff score of >2.0, *P* < 0.05) PICRUSt-predicted functional pathways in CDI and non-CDI samples considering untreated (A), fidaxomicin-treated (B), metronidazole-treated (C), and vancomycin-treated (D) samples. Download FIG S3, TIF file, 1.2 MB. Copyright © 2018 Lamendella et al. 2018 Lamendella et al. This content is distributed under the terms of the Creative Commons Attribution 4.0 International license.

### Fungal community structure comparisons.

**FIG 4:**
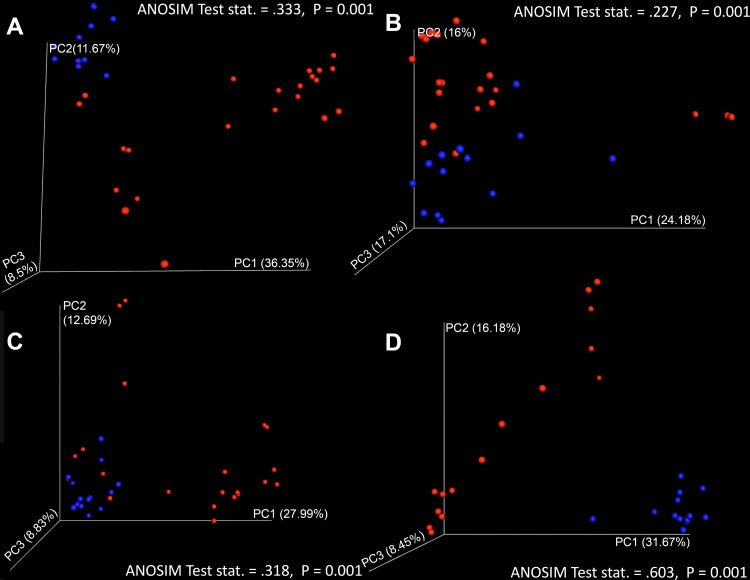
Three-dimensional principal-coordinate (PC) analysis plots were generated in QIIME 1.9.0 and visualized with EMPeror to Bray-Curtis distances between CDI and non-CDI samples in each treatment cohort (A, untreated; B, fidaxomicin; C, metronidazole; D, vancomycin) considering fungal taxon annotations. Here, we can visualize significant differential clustering between disease statuses when all treatment cohorts are included (ANOSIM *P* < 0.002).

In each antibiotic treatment cohort tested, there was no significant difference in observed species richness in any respective CDI versus non-CDI comparison (all two-sample *t* tests with Monte Carlo permutations, *P* > 0.05). Principal-coordinate analysis, however, revealed significant clustering of samples on the basis of CDI status when each respective antibiotic treatment was considered (ANOSIM fidaxomicin test statistic = 0.277 [*P* = 0.002], vancomycin test statistic = 0.603 [*P* = 0.001], metronidazole test statistic = 0.318 [*P* = 0.001], and untreated sample test statistic = 0.333 [*P* = 0.001]) (Fig. 4).

**FIG 5:**
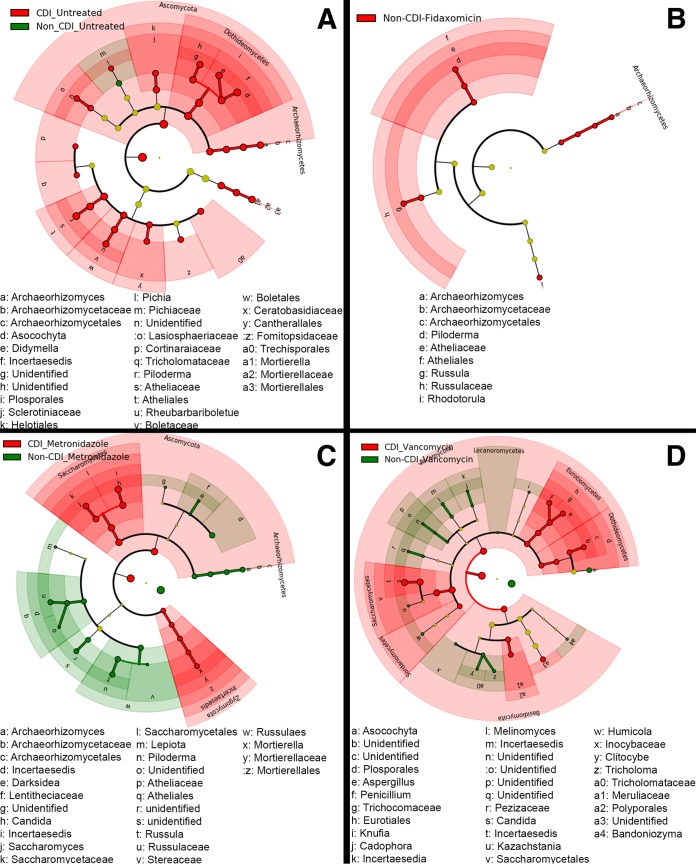
Cladogram plots were generated in Galaxy to visualize significantly enriched fungal taxa identified in CDI and non-CDI samples considering each treatment cohort separately (A, untreated; B, fidaxomicin; C, metronidazole; D, vancomycin).

Regarding paired cladogram LEfSe plots (Fig. 5), among untreated samples, the only enriched taxon in the non-CDI group was in the family *Pichiaceae* of the order *Saccharomycetales*; in the CDI cohort, a significantly increased relative abundance of fungal taxa was noted, including the phylum *Ascomycota*, the order *Pleosporales*, and the class *Dothideomycetes*. With metronidazole treatment, non-CDI samples were enriched with the family *Styracaceae*, the genus *Darksidea*, and the family *Lentitheciaceae*; among CDI samples, a relative abundance of the class *Saccharomycetes* and the phylum *Ascomycota* was observed. In the vancomycin treatment group, non-CDI samples were enriched with the genera *Cadophora*, *Bandoniozyma*, and *Clitocybe*, while CDI samples were enriched with the phylum *Ascomycota* and the order *Saccharomycetales*. For fidaxomicin-treated samples, non-CDI samples were enriched with nine taxa predominantly from *Archaeorhizomyces* compared to CDI samples. No fungal taxa were enriched in fidaxomicin-treated CDI samples. Paired LEfSe data are provided in Fig. S4.

10.1128/mSphere.00572-17.4 FIG S4  LEfSe plots were generated to display biomarker fungal taxa driving shifts in microbial community structure between CDI and non-CDI samples in untreated (A), fidaxomicin-treated (B), vancomycin-treated (C), and metronidazole-treated (D) samples. Download FIG S4, TIF file, 0.7 MB. Copyright © 2018 Lamendella et al. 2018 Lamendella et al. This content is distributed under the terms of the Creative Commons Attribution 4.0 International license.

### Bipartite co-occurrence networks.

**FIG 6:**
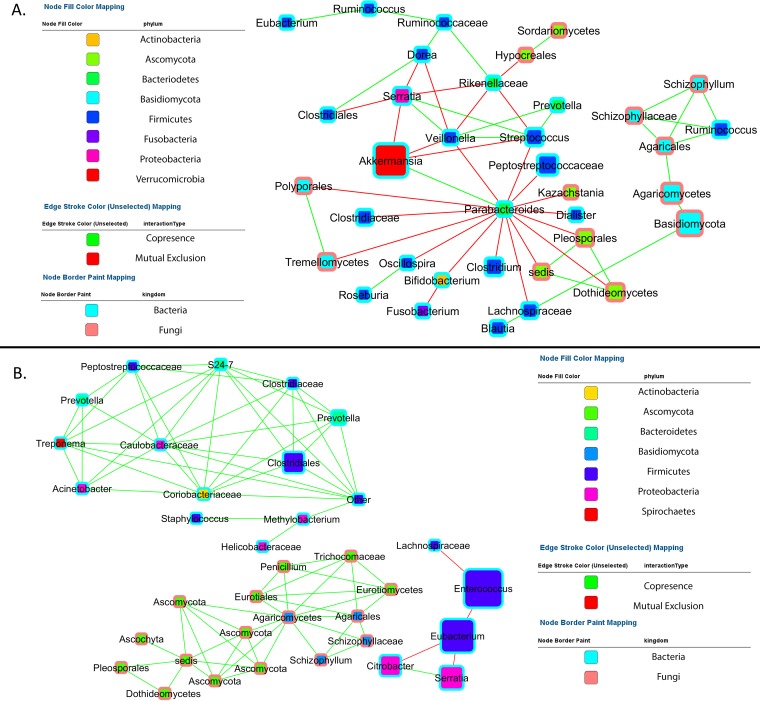
Bipartite co-occurrence networks were generated in CoNet and visualized in Cytoscape to display significant strong positive and negative co-occurring relationships between fungal and bacterial taxa in untreated CDI samples (A) and untreated non-CDI samples (B).

Networks for CDI samples not treated with antibiotics demonstrated predominantly negative interactions between a smaller number of potentially pathogenic taxa and a large variety of commensals, in keeping with a dysbiotic gut ecology. In untreated CDI samples, taxa of the genera *Akkermansia* were noted to have negative interactions with *Streptococcus* and *Veillonella* while having positive interactions with *Parabacteroides* bacteria, which themselves were negatively associated with *Clostridium*, *Bifidobacterium*, and *Lachnospiraceae* bacteria (Fig. 6A).

In metronidazole- and vancomycin-treated CDI samples, a relative abundance of *Saccharomyces* was noted, which had negative interactions with both *Ruminococcaceae* and *Coprococcus*, two organism groups that have a function in the production of short-chain fatty acids (Fig. S5A and B). In contradistinction, among fidaxomicin-treated CDI samples (Fig. S5C), several operational taxonomic units (OTUs) of *Saccharomyces* were noted to co-occur with each other. Further, a strong negative interaction between *Saccharomyces* and *Candida* was noted with fidaxomicin treatment of CDI samples. Additionally, with fidaxomicin treatment of CDI samples, *Saccharomyces* shared negative interactions with *Veillonella*, which is a biomarker of inflammatory diseases of the gut, including inflammatory bowel disease (17).

10.1128/mSphere.00572-17.5 FIG S5  Tandem co-occurrence networks were generated with CoNet and visualized in Cytoscape to display strong (Spearman’s rho = >|0.65|) positive (green edge) and negative (red edge) relationships between bacterial (blue outline) and fungal (red outline) taxa in fidaxomicin (A)-, metronidazole (B)-, and vancomycin (C)-treated CDI samples. Download FIG S5, TIF file, 1.4 MB. Copyright © 2018 Lamendella et al. 2018 Lamendella et al. This content is distributed under the terms of the Creative Commons Attribution 4.0 International license.

## DISCUSSION

In this study using fecal samples obtained from hospitalized patients with diarrhea, the three most commonly used *C. difficile*-directed antibiotics created reductions in alpha diversity. Beta diversity, as assessed with weighted UniFrac calculations, demonstrated compositions of dysbiotic states distinctive for the type of antibiotic used. Paired LEfSe analyses for both bacterial and fungal taxa further demonstrated the relative abundance of assemblages in CDI-positive stool samples that were distinctive for the different *C. difficile*-directed therapies studied. Regarding bacterial taxa, metronidazole treatment was associated with relative abundances of *Enterobacteriales* and *Citrobacter*, two common nosocomial pathogen groups. A similar LEfSe analysis result was obtained with fidaxomicin-treated CDI stool samples, with relative abundances of *Enterobacteriales* in addition to *Bacteroidia*. An interesting similarity in enriched taxa between vancomycin-treated CDI samples (*Peptostreptococcaceae*) and those not treated with antibiotics (*Clostridia* and members of the class *Bacteroidia*) was also observed. While untreated CDI-positive samples were consistently enriched with fungal taxa such as *Ascomycota*, *Pleosporales*, and *Dothideomycetes*, fidaxomicin treatment was associated with no enriched differentiating fungal taxa compared to untreated CDI stool samples.

Fidaxomicin is a water-insoluble macrocyclic compound isolated from the fermentation of *Dactylosporangium aurantiacum* (18), with its mechanism of action related to the inhibition of protein synthesis via interference with bacterial sigma factor subunits. This drug has a limited spectrum of antibacterial activity, with documented resistance to fidaxomicin observed in *Enterococcus* and *Bacteroides* species (19, 20), which would potentially improve CDI cure and recurrence rates by limiting off-target microbial effects commonly observed with other conventional antibiotics such as metronidazole and vancomycin.

Two large, prospective, multicenter, double-blind, randomized clinical trials (13, 21) have compared fidaxomicin to vancomycin; fidaxomicin demonstrated noninferiority to vancomycin in terms of cure rates, with one of these studies demonstrating a lower rate of recurrent infections when patients with the potentially virulent NAP-1 strain of *C. difficile* were excluded. Further, fidaxomicin-treated patients were not observed to have higher incidences of treatment-related adverse events. Additional studies (15) suggest that for patients receiving concomitant antibiotics in addition to *C. difficile*-directed therapy, fidaxomicin-treated CDI patients were more likely than vancomycin-treated patients to achieve a cure (90% versus 74.7% [*P* = 0.005]). Despite its higher cost, there are data (22) that suggest that fidaxomicin may be more cost effective than vancomycin because of lower recurrence rates, leading to lower overall treatment costs. Several drug mechanisms (23) may account for the above-described clinical findings, including a prolonged postantibiotic effect, suppression of exotoxin production and sporulation, and the inhibition of transcription inhibitors. Whether these *in vitro* observations are clinically relevant is unclear, though previous studies (24) with somewhat limited evaluations of bacterial diversity in fidaxomicin-treated patients suggest that certain consortia associated with gut health were better preserved with this antibiotic, as demonstrated by a lesser degree of reduction of organisms belonging to *Bacteroides* and *Prevotella*. Despite these potential advantages, fidaxomicin has been largely relegated to the outpatient management of milder forms of CDI; because of its expense and the absence of a parenteral form of the drug, it is less frequently used among inpatients than metronidazole and vancomycin and its role among inpatients remains somewhat limited.

In this study, aspects of antibiotic therapy beyond the spectrum of antimicrobial activity were investigated. One such line of inquiry involved the use of imputed functional metagenomics with PICRUSt, correlating microbial community abundances, with predicted antibiotic-induced changes, with resultant community functions that might potentially contribute to the pathogenesis of CDI. Antibiotic-untreated CDI stool samples demonstrated an enrichment in pathways related to LPS metabolism that were enriched to a degree statistically similar to that of antibiotic-treated CDI samples. This, as well as enriched pathways related to arachidonic acid, may represent toxin-independent mechanisms for the colitis that characterizes clinically symptomatic CDI (25). The latter observation is of particular interest, given previous reports (26, 27) describing how the large clostridial toxins can stimulate the production of proinflammatory leukotrienes and prostaglandins from gut-associated immune components such as neutrophils. The production of arachidonic acid by bacterial communities may add to the severity of colitis through a mechanism distinct from the inflammation caused by an immune response to *C. difficile*. An additional enriched pathway in our untreated CDI samples involved those related to the production of novobiocin. It is known that bacteria and fungi can produce xenobiotic compounds that can have an antibiotic-like effect, and our group has recently described this phenomenon in the mycobiome of human patients with CDI (16). Though metatranscriptomics and animal modeling are required for confirmation, these observations may provide a novel insight into the high persistence and recurrence rates (25 to 50%) associated with CDI therapy (28). *C. difficile* is highly selectively advantaged to thrive in gut environments with diminished bacterial population density and diversity, and this gut dysbiosis may be perpetuated if there is a relative abundance of xenobiotic-producing organisms. Considering that antibiotics are the most frequent cause of this dysbiosis and considering the collateral effects of *C. difficile*-directed antibiotics on off-target bacteria, this convergence of iatrogenic and microbial factors may indicate that CDI treatment failures are less related to inefficacy of antibiotics against *C. difficile* and that disease persistence and recurrence may be more related to enriched bacterial and fungal communities that resist the restoration of a eubiotic state.

In CDI samples, metronidazole and fidaxomicin were both associated with enrichment of pathways related to glycan synthesis and metabolism. Glycan metabolism has a major influence on the composition of the gut microbiome (29), and the ability of certain organisms to utilize glycans consumed by the host, as well as luminal versus mucosal spatial distribution of glycans and glycan-metabolizing microbes, may have a significant effect on the development of dysbiotic states. With the loss of nonpathogenic, mucosa-adherent organisms because of antibiotics that occurs in CDI, the greater availability of glycans (30) may allow pathogenic organisms such as *C. difficile* to expand their population through an incipient nutritional niche.

Vancomycin treatment was associated with enrichment of pathways for isoquinoline compounds in both CDI and non-CDI stool samples; the enrichment of this pathway regardless of CDI status suggests that this observation represents an antibiotic-induced community function. These heterocyclic aromatic compounds are used in the production of antimicrobial agents (31), including antifungal agents (32).

The use of PICRUSt for predictive metagenomic profiling has been demonstrated to yield useful information regarding community function and at a cost much lower than that of deep shotgun metagenomic sequencing. However, predictive metagenomic profiling has important limitations (33). Compared to deep sequencing, PICRUSt is limited in the extent to which sequenced genomes are available for the most abundant species in the community of interest, an important limitation for the assessment of imputed function (34). To address some of these limitations, we report the weighted NSTI scores to summarize the extent to which predicted functions are related to sequenced genomes.

The role of the mycobiome in CDI is a topic that is infrequently investigated in part because of the technical challenges of internal transcribed spacer (ITS) sequencing and the limitations of reference databases for fungal taxa, leaving many fungal taxa incertae sedis. The observation that fungal taxa produce antibiotic-like compounds requires further study to identify whether this phenomenon is a frequent and clinically relevant occurrence in human CDI, though its potential relationship to CDI is intriguing. The concept of transkingdom interactions between bacteria and fungi leading to an advantage for *C. difficile* has seminal data to support it. Using a coculture technique, van Leeuwen and colleagues (35) demonstrated that the presence of *Candida albicans* allowed *C. difficile* to survive in an aerobic environment, although the latter is an obligate anaerobe. Interestingly, the production of *p*-cresol by *C. difficile* prevented the development of candidal hyphae, which limits the virulence of *C. albicans*, suggesting that this bacterial-fungal interaction enhances the virulence of *C. difficile* while limiting *Candida* pathogenicity. In another study, Jawhara and colleagues used wild-type and Gal3^−/−^ mice in a dextran sulfate-sodium (DSS) model of colitis with the addition of *Candida* to the mouse alimentary tract only after the onset of colitis (36). The presence of *Candida* augmented the degree of inflammation noted in DSS-treated colons in this knockout model.

On the basis of network analyses, a potential advantage associated with fidaxomicin treatment would include relative abundances of several *Saccharomyces* OTUs that were associated with a decrease in candidal abundance. *Saccharomyces* enrichment has been studied in combination with antibiotics as an intervention to reduce recurrent CDI (37), as well as to prevent the development of CDI (38). Additionally, the negative correlation between *Saccharomyces* and *Veillonella* in fidaxomicin-treated samples, coupled with a lesser degree of *Ruminococcaceae* and *Coprococcus* depletion with fidaxomicin treatment, may suggest a shift in microbial community structure that is less inflammatory than with other *C. difficile*-directed therapies.

Future work will focus on applying these data derived from human CDI patients to an animal model to evaluate the role of fungal taxa in the pathogenesis of CDI. In conclusion, the present study provides evidence that CDI includes interactions from both bacterial and fungal microbial communities, with the potential that these interactions reinforce the dysbiosis that could be an important determinant of recurrent and persistent CDI. The resultant changes in community composition and function suggest that antifungal therapy should be evaluated as an adjunct to *C. difficile*-directed antibiotics, though additional data are needed to further establish the plausibility of this suggestion. *C. difficile* antibiotic therapies have distinct gut ecological impacts, with a possible advantage for fidaxomicin in terms of a lesser relative abundance of pathogenic fungi and with imputed community functions that may promote a diminished severity of inflammation.

## MATERIALS AND METHODS

This study was an investigator-initiated, industry-sponsored project performed with the approval of the Penn State Milton S. Hershey Medical Center (PSHMC) Institutional Review Board (IRB). Before stool sample collection, each patient signed an IRB-approved consent form.

### Patients.

Twenty inpatients admitted to the senior author’s institution (PSHMC) for the treatment of various medical ailments were enrolled in this study between March and September 2016. Patients who were at least 18 years old were eligible, with no maximum age. Patients receiving chemotherapy within 60 days of potential enrollment, those with inflammatory bowel disease, those with a history of a positive *C. difficile* test within 60 days of potential enrollment, those empirically started on *C. difficile*-directed antibiotics before stool sample testing, and those within 30 days of a mechanical bowel preparation were ineligible for inclusion.

Only diarrheal stool samples were collected for analysis in this study. As part of routine clinical care, each patient with a clinical suspicion of CDI had a stool sample sent by the treating physician to the PSHMC clinical microbiology lab for *C. difficile* testing with a commercially available nucleic acid amplification test (Illumigene *C. difficile*; Meridian Bioscience, Cincinnati, OH) designed to detect a highly conserved sequence in the *tcdA* gene. During the enrollment period, *C. difficile*-positive and -negative stool samples were preserved, after clinical testing, in a −80ºC freezer until the patients consented to inclusion in this study. Stool samples collected for research purposes were sent for CDI testing before the administration of CDI therapy.

Once all of the stool samples were collected, they were processed, in duplicate, in the following manner by treating CDI and non-CDI samples during separate but consecutive days to limit the number of thawing-refreezing cycles while performing all volume transfers under anaerobic conditions. One gram of each stool sample was thawed to room temperature, with each sample diluted in 0.45× brucella broth composed of 4 ml of brucella broth supplemented with vitamin K and hemin, which was further diluted with 5 ml of reduced minimal salt buffered water supplemented with sodium thioglycolate and l-cysteine. The diluted stool sample was vortexed and then transferred with a 1-ml syringe into eight separate brucella broth aliquots (1:10), yielding a total of 80 stool sample cultures (10 mg/ml feces), half of which were *C. difficile* positive. Of these 80 stool sample cultures, 20 were not treated with antibiotics, while the rest were treated with one of three antibiotics by preparing stocks at 100× the target fecal concentrations as follows: (i) vancomycin (*n* = 20), prepared with 1 g of vancomycin in 10 ml of dimethyl sulfoxide (DMSO) for a fecal concentration of 1,000 µg/ml; (ii) metronidazole (*n* = 20), prepared with 0.1 g of metronidazole in 10 ml of DMSO for a fecal concentration of 10 µg/ml; (iii) fidaxomicin (*n* = 20), prepared with 1 g of drug in 10 ml of DMSO for a fecal concentration of 1,000 µg/ml (39–41). After each stool sample was rocked on a platform rocker (VWR, Radnor, PA) at 20 rpm at 37°C for 1 h, 100 µl of an antibiotic solution was added to samples as appropriate and antibiotic-treated cultures were incubated with rocking at 37°C for 24 h. These treated cultures were then frozen at −80°C for transfer to the Lamendella laboratory at Juniata College (Huntingdon, PA) for sequencing.

In addition, two “pooled” diluted stool samples were created, each consisting of 1 ml of each of the 10 diluted stool samples from each of the two study cohorts; 1 ml from each pooled cohort sample was then diluted 1:10 into four brucella broth cultures, yielding 8 pooled samples in total, 4 from the non-CDI cohort and 4 from the CDI cohort. Two of these eight samples were left untreated, with the remaining samples treated with vancomycin, metronidazole, and fidaxomicin as previously described. Untreated and treated samples were then frozen at −80°C and shipped on dry ice to the Lamendella laboratory for further processing.

### Bacterial community profiling.

DNA was extracted from approximately 680 μl of a homogenized fecal solution (*n* = 176) with a Mo Bio PowerFecal DNA extraction kit in accordance with the manufacturer’s instructions (Mo Bio Laboratories, Carlsbad, CA). Illumina iTag PCR mixtures (25 μl) contained approximately 5 to 10 ng of template DNA with a final concentration of 1× PCR buffer, 0.8 mM deoxynucleoside triphosphates, 0.625 U of *Taq* polymerase, 0.2 μM barcoded 515F forward primer, and 0.2 μM Illumina 806R reverse primer per reaction mixture (53). Each PCR was carried out with an MJ Research PTC-200 thermocycler (Bio-Rad, Hercules, CA). Pooled PCR products were gel purified with a QIAquick gel extraction kit (Qiagen, Frederick, MD) and quantified with a Qubit 2.0 fluorometer (Life Technologies, Inc., Carlsbad, CA).

Before submission for sequencing, libraries were quality checked with the 2100 Bioanalyzer DNA 1000 chip (Agilent Technologies, Santa Clara, CA). Library pools were size verified with the Fragment Analyzer CE (Advanced Analytical Technologies Inc., Ames, IA) and quantified with a Qubit high-sensitivity double-stranded DNA kit (Life Technologies, Inc., Carlsbad, CA). Purified libraries were sent to Laragen Inc. (Culver City, CA) for sequencing with an Illumina MiSeq V2 500 cycle kit cassette with 16S rRNA gene library sequencing primers set for 250-base paired-end reads.

Sequences were first processed with VSEARCH v1.11.1 to merge forward and reverse reads with a minimum overlap set to 200 bp and then truncated at a length of 210 bp (42). Reads were quality filtered with a maximum error rate of 0.5% with USEARCH v7 (43). Quality-filtered sequences were then analyzed with QIIME 1.9.0 (44). Chimeric sequences were identified by VSEARCH *de novo*-based chimera checking, and a total of 5,870,263 sequences, 11,103 OTUs, and 173 samples remained after quality filtering and chimera checking. Open reference OTUs were picked by using the VSEARCH v1.11.1 algorithm, and taxonomy assignment was performed by using the Greengenes 16S rRNA gene database (13-5 release, 97%) (45). Clustered taxa were compiled into a biological observation matrix OTU table.

The unrarified OTU table underwent singleton removal and normalization with the metagenomeSeq Cumulative Sum Scaling (CSS) algorithm for beta diversity analysis (46, 47). Alpha diversity indices, including observed species and PD whole tree, were generated from an unrarified OTU table with a maximal sampling depth of 8,300, a step size of 830, and 20 iterations at each step. Alpha diversities were then collated, plotted, and compared by considering all of the indices generated. Alpha diversity was compared in antibiotic experimental cohorts (CDI versus non-CDI) with a two-sample *t* test and nonparametric Monte Carlo permutations (*n* = 999). Principal-coordinate analysis plots, ANOSIM tests for significance (α < 0.05), and distance boxplots were calculated by using a weighted UniFrac distance matrix generated from a CSS-normalized OTU table in QIIME 1.9.0.

PICRUSt functional predictions were generated from a closed-reference OTU table generated in QIIME 1.9.0 by using the KEGG orthology reference database. 16S rRNA gene copy number corrections were conducted on all functional predictions. LEfSe was also used to identify taxonomic, as well as predicted functional, biomarkers in the CDI and non-CDI cohorts (48). Genus level relative abundances, as well as PICRUSt-predicted functions, were multiplied by one million and formatted as previously described (46). Comparisons were made with antibiotic treatment (CDI versus non-CDI) as the main categorical variable. An alpha level of 0.05 was used for Kruskal-Wallis tests, and an LDA cutoff score of 1 was used to display the enriched predicted functions in each cohort, whereas the top 20 predicted taxa in each respective cohort were displayed for 16S rRNA gene and ITS comparisons. Features were plotted on a logarithmic scale according to the treatment group with which they were significantly associated.

### Fungal community analysis.

The ITS1 region was amplified in 25-μl PCR mixtures by using the ITS1 forward and Illumina barcoded ITS2 reverse primers designed to target the variable region as described by Smith and Peay (49). The thermocycling conditions on an MJ Research PTC-200 thermocycler (Bio-Rad, Hercules, CA) were as follows: 40 cycles of 94°C for 30 s, 52°C for 30 s, and 68°C for 30 s; 68°C for 7 min; and a 4°C hold. Pooled PCR products were gel purified with a QIAquick gel extraction kit (Qiagen, Frederick, MD) and quantified with a Qubit 2.0 fluorometer (Life Technologies, Inc., Carlsbad, CA). Purified libraries were then sequenced with the NextSeq Mid Output kit with single-end 150-bp sequencing on the Illumina NextSeq at the University of California Davis Genomics Sequencing Core.

A total of 47,148,427 ITS reads were trimmed with Trimmomatic (50) to remove low-quality regions. Reads were then truncated to a length of 145 bp to retain sequences with *Q* scores of >29 with quality filtering at a maximum error rate of 1.0% with VSEARCH v1.11.1 (42). A total of 132 samples containing 37,867,398 sequences were used for downstream analysis. OTUs were picked with the open-reference UCLUST algorithm (43) in QIIME 1.9.0 at the default OTU threshold of 0.97, and singleton sequences were discarded (44). Taxonomy was assigned by using the BLAST option in the assign_taxonomy.py script against version 7 of the UNITE fungal ITS database (51) with the maximum E value set to the default of 0.001. All taxa with no BLAST hits were removed from the OTU table for downstream analysis. Alpha diversity observed species indices were generated from an unrarified OTU table with a maximum sampling depth of 16,000, a step size of 1,600, and 20 iterations at each step. Observed species richness in antibiotic-treated experimental cohorts (CDI versus non-CDI) was then compared with a two-sample *t* test and nonparametric Monte Carlo permutations (*n* = 999). PD whole-tree metrics were not utilized, as a reference tree file cannot be generated for fungal ITS data sets.

### Network analysis.

Co-occurrence networks were constructed from bacterial and fungal level 6 (genus level) summaries and visualized in Cytoscape 3.3.0 with the CoNet plugin (52). For an OTU to be included in the network, it needed to occur in at least 50% of the samples and have a Spearman’s rho threshold of at least |0.65|. Spearman’s correlations were paired with two dissimilarity measures, Bray-Curtis and Kullback-Leibler, to calculate the distances between nodes. An edge selection of 50 was utilized for all generated networks. All three statistical measures ensured minimal significant correlations due to outliers, matching zeros, or error in data composition. To adjust for multiple testing corrections, the Benjamini-Hochberg correction was used to adjust *P* values in the last network processing step. Bacterial and fungal taxa were labeled down to the lowest identified taxonomic ranking, and all unassigned taxa were removed from the networks.

### Data availability.

All sequence data and metadata are available through NCBI's Sequence Read Archive under BioProject no. PRJNA427597 (SRP127616).
