# Supplementary figures and images for: Antibiotic Treatments for Clostridium difficile Infection Are Associated with Distinct Bacterial and Fungal Community Structures
Source: mSphere. 2018 Jan 10;3(1):e00572-17. doi: 10.1128/mSphere.00572-17 (PMC5760750; doi:10.1128/mSphere.00572-17)

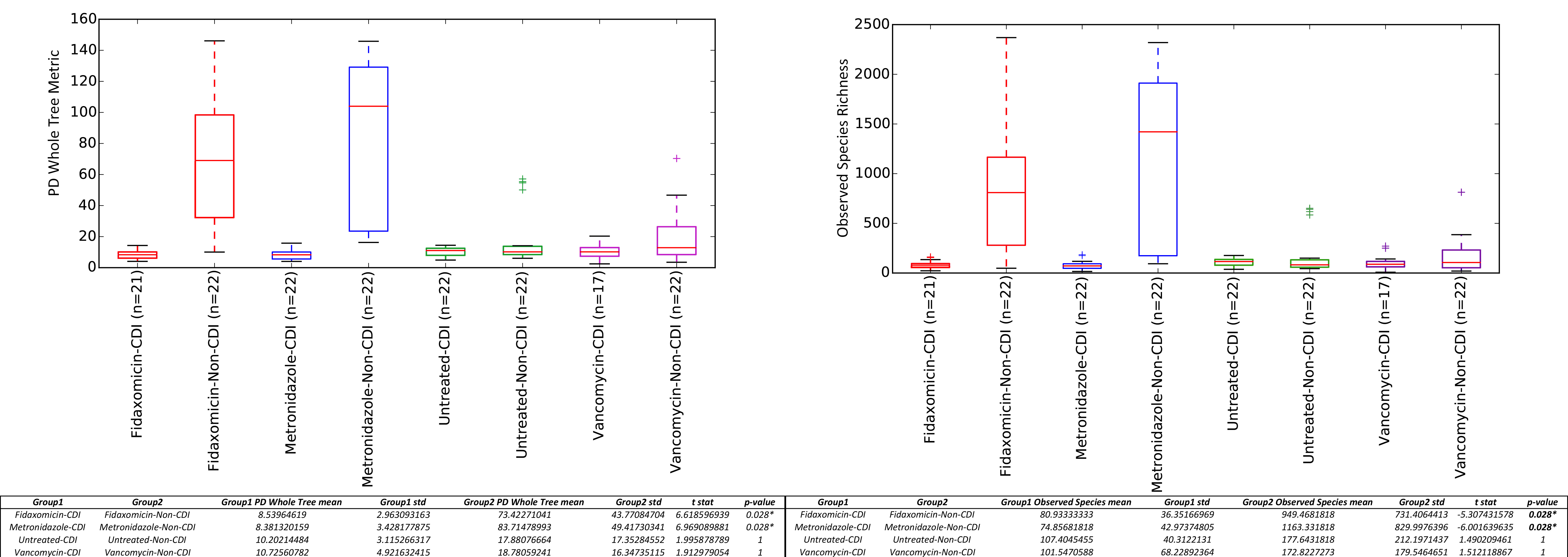

Supplement: FIG S1 [file sph001182452sf1.jpg]

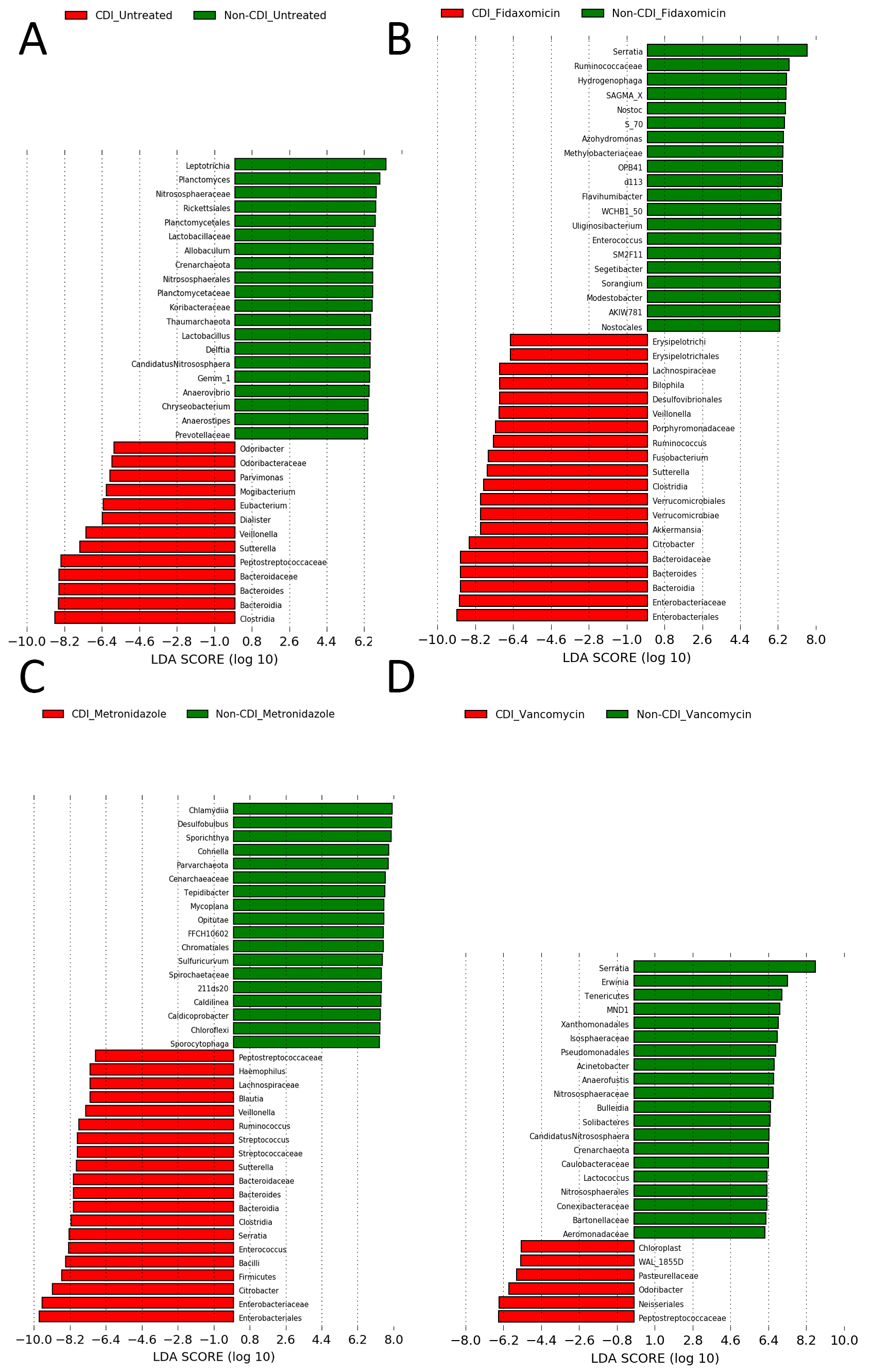

Supplement: FIG S2 [file sph001182452sf2.tif]

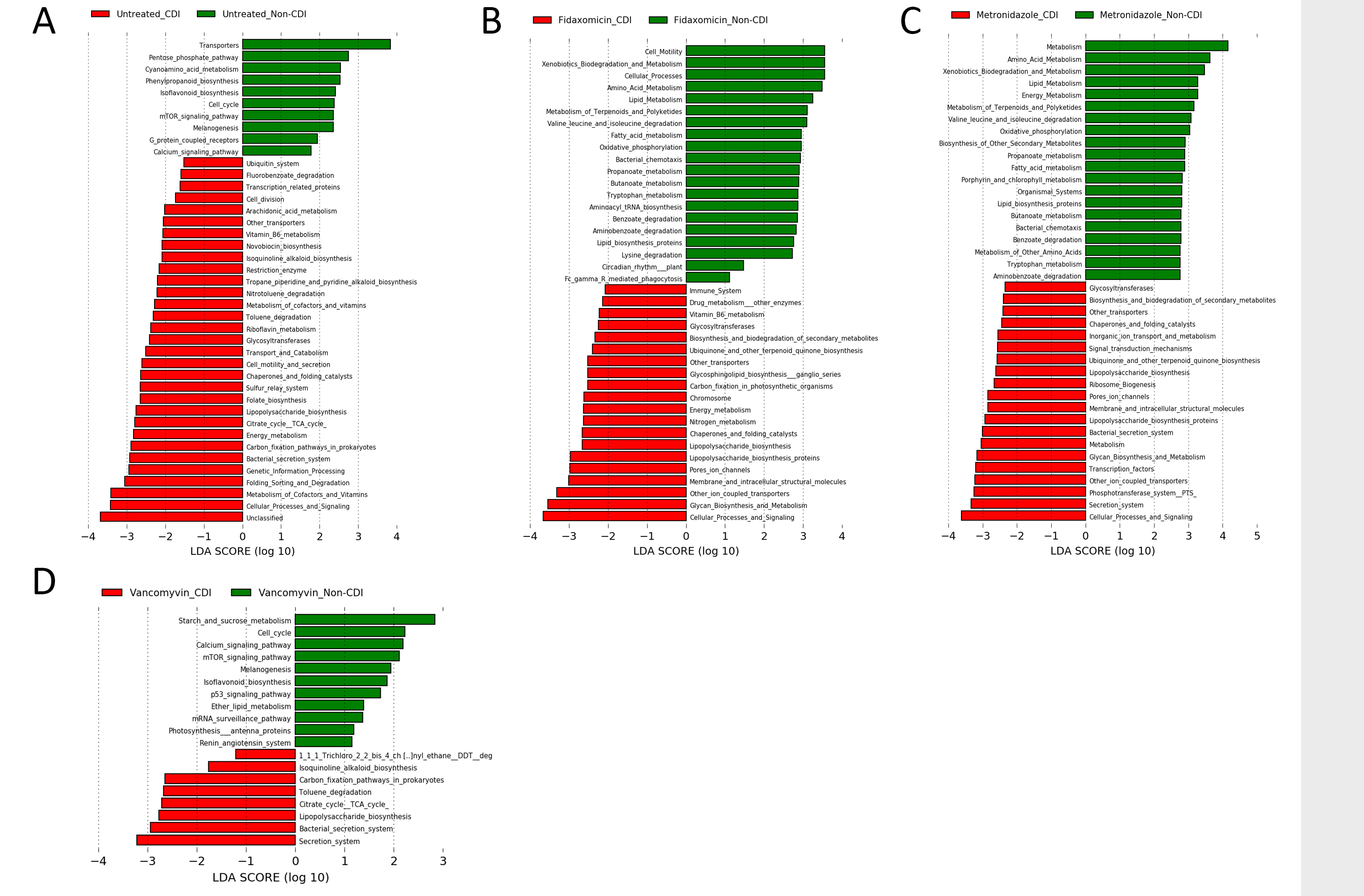

Supplement: FIG S3 [file sph001182452sf3.tif]

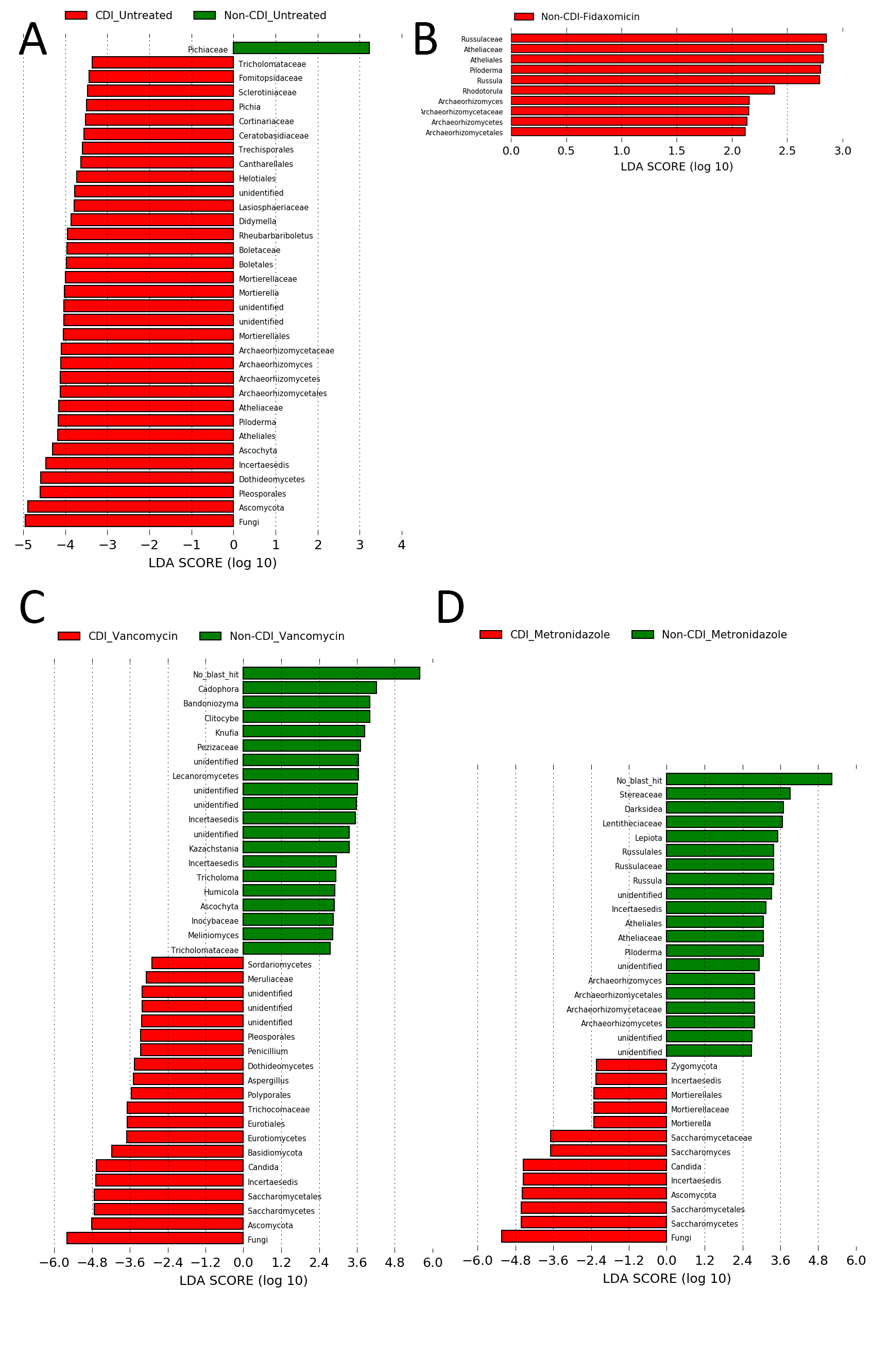

Supplement: FIG S4 [file sph001182452sf4.tif]

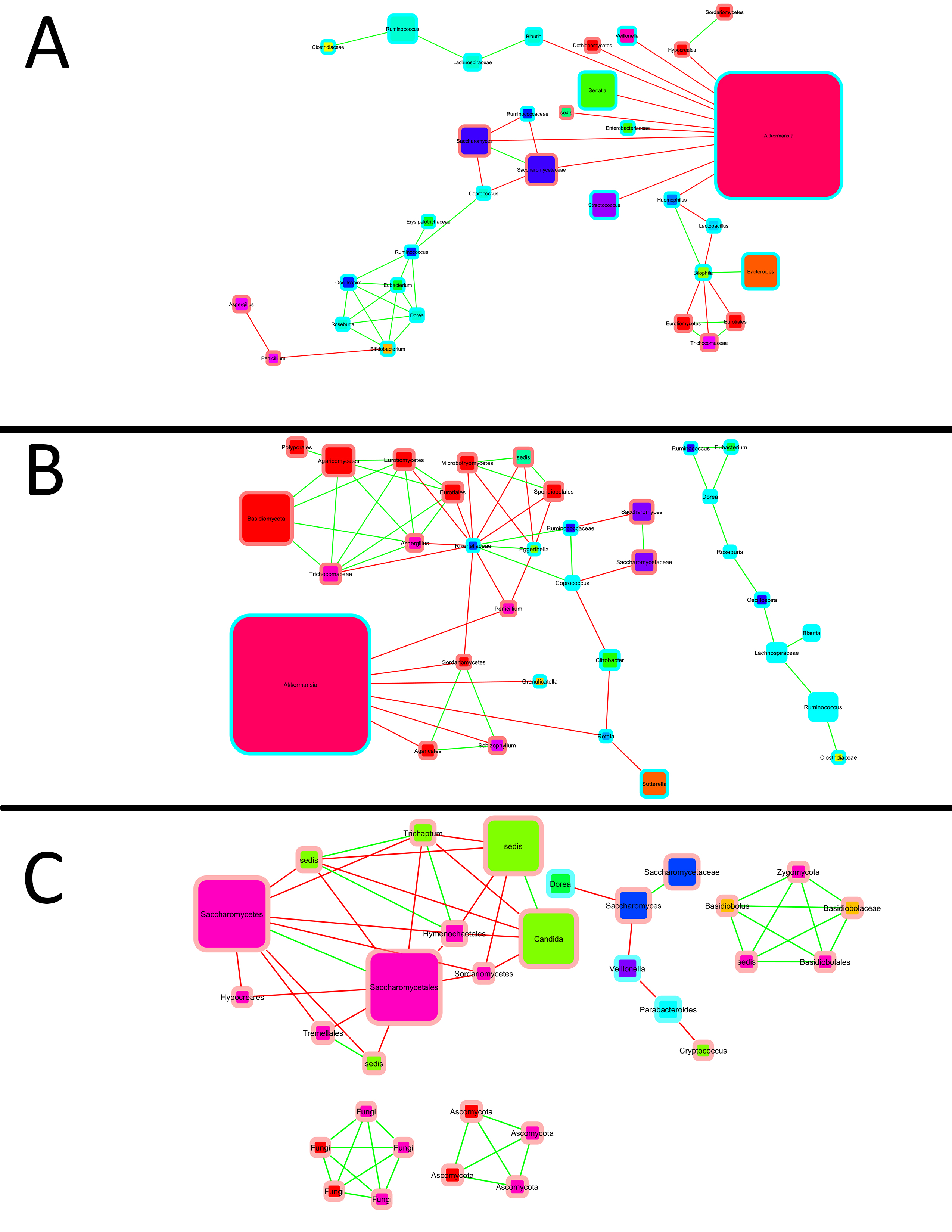

Supplement: FIG S5 [file sph001182452sf5.tif]
